# Non-Occlusive Mesenteric Ischemia in Children With Diabetic Ketoacidosis: Case Report and Review of Literature

**DOI:** 10.3389/fendo.2022.900325

**Published:** 2022-07-14

**Authors:** Giulio Frontino, Raffaella Di Tonno, Valeria Castorani, Andrea Rigamonti, Elisa Morotti, Federica Sandullo, Francesco Scialabba, Francesca Arrigoni, Riccardo Foglino, Benedetta Dionisi, Chiara Irene Carla Ferri, Salvatore Zirpoli, Graziano Barera, Franco Meschi, Riccardo Bonfanti

**Affiliations:** ^1^ Department of Pediatrics, IRCCS San Raffaele Hospital, Milan, Italy; ^2^ Diabetes Research Institute, IRCCS San Raffaele Hospital, Milan, Italy; ^3^ Pediatric Radiology and Neuroradiology, Children’s Hospital “V. Buzzi”, Milan, Italy; ^4^ Vita-Salute San Raffaele University, Milan, Italy

**Keywords:** non-occlusive mesenteric ischemia (NOMI), DKA (diabetic ketoacidosis), insulin pump (CSII: continuous subcutaneous insulin infusion), acute kidney injury, hyperosmolar (hyperglycemic) coma, T1DM (type 1 diabetes mellitus)

## Abstract

**Introduction:**

Despite the use of technology, recurrent diabetic ketoacidosis (DKA) prevention remains an unmet need in children and adolescents with T1D and may be accompanied by life-threatening acute complications. We present a rare case of non-occlusive mesenteric ischemia (NOMI) with overt manifestation after DKA resolution and a discussion of recent literature addressing DKA-associated NOMI epidemiology and pathogenesis in children and adolescents.

**Case Presentation:**

A 13-year-old female with previously diagnosed T1D, was admitted at our emergency department with hypovolemic shock, DKA, hyperosmolar state and acute kidney injury (AKI). Mildly progressive abdominal pain persisted after DKA correction and after repeated ultrasound evaluations ultimately suspect for intestinal perforation, an intraoperative diagnosis of NOMI was made.

**Conclusion:**

The diagnosis of DKA-associated NOMI must be suspected in pediatric patients with DKA, persistent abdominal pain, and severe dehydration even after DKA resolution.

## Introduction

Diabetic ketoacidosis (DKA) is the most serious life-threatening acute complication of Type 1 Diabetes (T1D). (1) DKA may present as the first manifestation of T1D, but may also occur occasionally during the course of the disease, or, more rarely, become a recurrent problem. DKA commonly presents with a short history of symptoms developing over a few weeks. Diagnosis is based on standard biochemical (hyperglycemia and metabolic acidosis) and clinical (dehydration, nausea, vomiting) signs. Additionally, abdominal pain is frequently observed, especially in patients with severe metabolic acidosis. (2)

Although the cause of gastrointestinal involvement has not been fully elucidated, delayed gastric emptying, paralytic ileus, electrolyte disturbances and metabolic acidosis may be involved (3, 4). A lack of resolution within the first 24 hours of treatment should prompt investigation for other causes. (3)

We therefore present a rare case of non-occlusive mesenteric ischemia (NOMI) that presented as mild persistent abdominal pain in the context of progressively resolved severe DKA. An appraisal of recent literature regarding epidemiology and physiopathology of DKA-associated NOMI in children and adolescents will also be provided.

## Case Report

A 13-year-old female with previously diagnosed T1D, was admitted to the emergency department presenting incoercible vomiting since the previous day, oligo-anuria and deteriorating state of consciousness. The girl had been diagnosed with autoimmune T1D at the age of 9 years (no DKA at onset). Past medical history was unremarkable. Sensor-augmented pump therapy (SAP) was started, and her follow-up was characterized by suboptimal glucose control (latest HbA1c of 60 mmol/mol).

**Figure 1:**
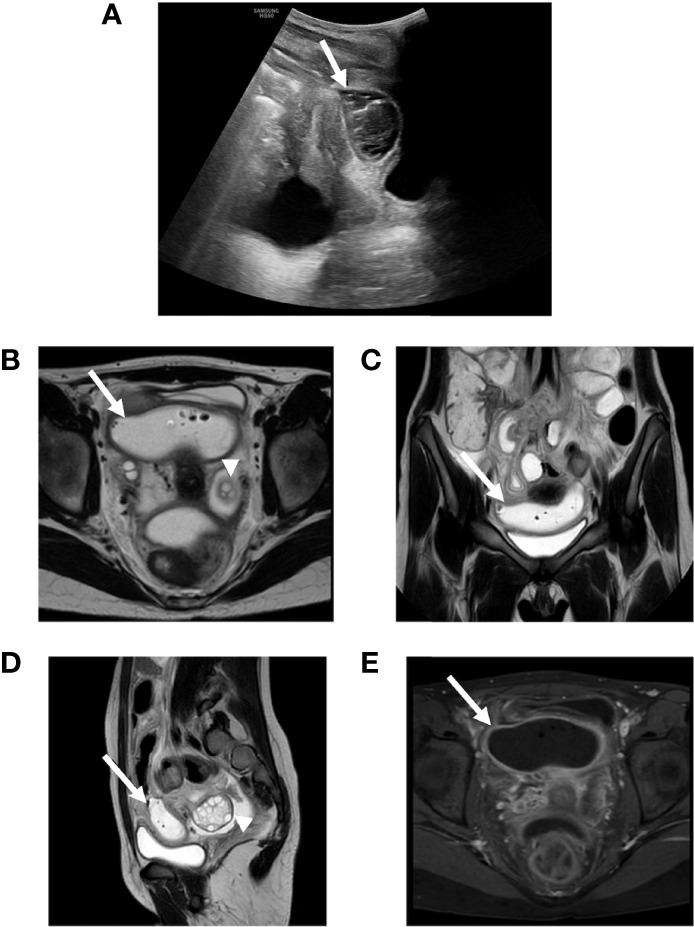
**(A)** Abdomen ultrasound showing an encapsulated abscess between bladder and uterus (arrow). **(B–E)** Abdomen MRI showing a pelvic large fluid-filled abscess (arrows) with air bubbles such as for bowel perforation (**B–D**: T2 weighted) with parietal contrast enhancement (**E**: T1 weighted) and normal adnexal region (arrowheads).

On admission, the child presented in hypovolemic shock with an altered state of consciousness (GCS 12) without evident focal neurological signs, pale skin and Kussmaul respiration. Her physical examination was unremarkable except for decreased and tachycardic distal pulses, cool extremities, prolonged capillary refill (> 2 sec). Venous blood gas analysis and lab tests showed severe DKA, hyperosmolar state, and acute kidney injury (AKI) with hyperkaliemia, confirmed at ECG analysis (**Table 1**). Two boluses of normal saline were infused (10 mL/kg each) with improvement in vital parameters. No bicarbonates were administered. The patient was then transferred to the intensive care unit (ICU) and DKA correction was carried out according to the most recent guidelines with progressive resolution of the DKA, hyperosmolar state, AKI, and electrolyte imbalances. A brain CT excluded cerebral edema. (2) On arrival the patient’s pump controller showed that the patch pump had expired. Of note, the patch pump is designed to shut-off automatically after 72h, after sounding alarms to alert the user to change the pump when approaching expiration. The child later admitted to not having changed the expiring patch pump while at her grandparents’ due to her not having any replacements and in fear of her mother’s anger. When DKA resolved, the patient was transferred to the pediatrics department and her SAP was reinstated. Since admission she referred modest abdominal pain that had persisted throughout DKA treatment. No other signs or symptoms were present apart from slightly loose stools which were collected for microbiological testing. An abdominal ultrasound only showed slight thickening of the ileal wall. On day 2 since DKA resolution, Clostridium difficile toxin tested positive and oral vancomycin treatment was started. On day 4, follow-up US only confirmed signs of enterocolitis. On day 5, abdominal symptoms and signs worsened alongside onset of fever and elevation of inflammatory indices (CRP 145 mg/L, normal range <6 mg/L). Thus, abdominal ultrasound was repeated, documenting an uneven multi-chambered area of 8x3 cm with a small aerial component (**Figure 1**). Treatment with metronidazole was started and the patient was transferred to the city’s center of reference for pediatric surgery. Abdominal magnetic resonance imaging revealed a large, fluid-filled pelvic abscess with air bubbles suspect for bowel perforation, with parietal contrast enhancement (**Figure 1**). Therefore, an urgent ileal resection (44 cm) was performed, and NOMI was diagnosed intraoperatively. Antibiotic treatment with piperacillin-tazobactam and gentamicin was administered for 7 days, and treatment with metronidazole and vancomycin was continued for 10 days. The patient was discharged after 3 weeks of transition from parenteral to enteral feeding. Pre-discharge abdominal ultrasound resulted normal without signs of inflammation or free abdominal fluid.

**Table 1:** Lab values.

Lab Values		Reference range
**ph** **HCO (mmol/L)** **pCO2 (mmHg)** **Base Excess (mmol/L)** **Anion Gap(mmol/L)** **Lactate (mmol/L)** **Blood Glucose (mg/dl)** **Osmolality (mOsm/kg)** **Creatinine (mg/dl)** **Sodium (mEq/L)** **Potassium (mEq/L)**	6.9 3 15 -29.9 40 6.39 1440 443 4.08 132.9 7.75	7.35-7.45 22-26 32-45 -2-3 12+/-4 0.7-2.1 60-100 275-295 0.50-1.10 135.0-148.0 3.50-5.00

## Discussion

Despite a recent Italian study has shown a decrease in the frequency of DKA at T1D diagnosis in children during the last years, DKA frequency remains unacceptably high. (4) Data on the incidence of recurrent DKA in children in Italy are less consistent. Retrospective study conducted in 29 Italian diabetes centers from November 2011 to April 2012 showed an incidence of secondary DKA of 2.4 events/100 py and tended to increase with age. (5) Lower socioeconomic status is associated with a higher risk of DKA and a higher rate of diabetes-related complications. (4, 6–9) The risk of DKA in children after diagnosis of T1D is usually resulting from intentional or inadvertent insulin omission, or sometimes associated with intercurrent illness and increased insulin requirement. (10–12) The classic clinical signs of DKA include polyuria, polydipsia, polyphagia, and weight loss, but they may progress rapidly to vomiting, abdominal pain, dehydration, weakness, and lethargy. Abdominal pain and ileus can result from potassium depletion, acidosis, and poor splanchnic perfusion. (13) These symptoms and signs have been described in 40-75% of the cases of DKA and typically resolve during the first 24 hours of treatment, after administration of fluids and insulin. (2) In the initial phase of DKA abdominal pain may be severe enough to mimic an acute abdomen. Surgery is warranted in 6% of adult cases (mainly for acute cholecystitis or appendicitis). (3, 14).

### The Pathogenesis of DKA and NOMI

**Figure 2:**
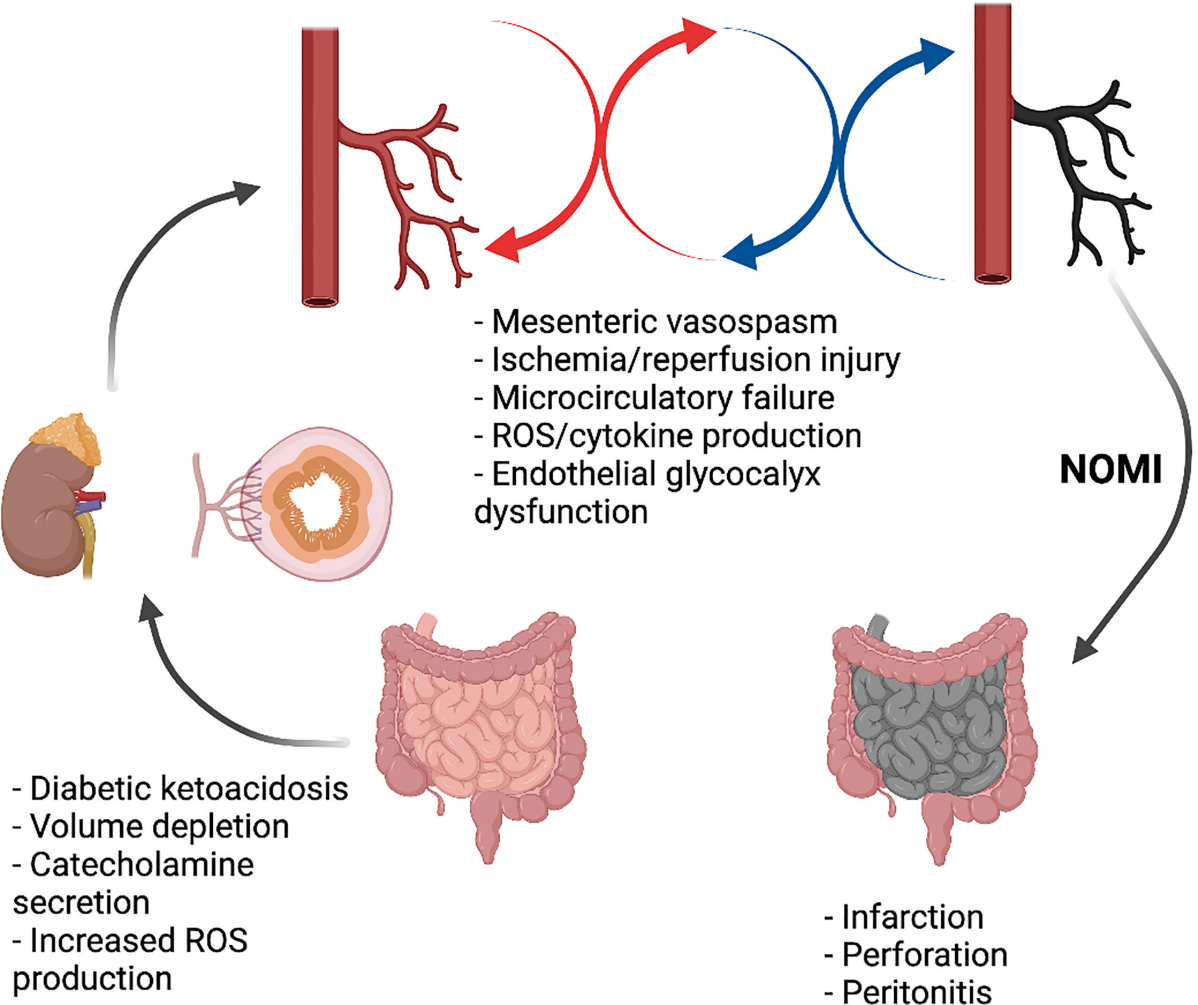
Non-occlusive mesenteric ischemia in DKA. Created with BioRender.com.

DKA is characterized by relative or absolute insulin deficiency and increased levels of counterregulatory hormones, such as glucagon, adrenaline, cortisol, growth hormone, and proinflammatory cytokines. (15) High plasma levels of counterregulatory hormone leads to gluconeogenesis and glycogenolysis with increased glucose production and decreased peripheral glucose utilization. This causes hyperglycemia, hyperosmolality, increased lipolysis, and ketogenesis. (13) Glucose-induced osmotic diuresis leads to vascular volume depletion, severe dehydration and low cardiac output; moreover, the high catecholaminergic expression may induce hypoperfusion. Therefore, a physiological mechanism is established to maintain the perfusion of vital organs at the expense of mesenteric perfusion. A mismatch between supply and demand develops in the intestine, due to persistent mesenteric vasoconstriction, resulting in reduced blood flow and oxygen supply to the intestine, particularly to the vulnerable superficial mucosa. (16) This represents the main pathological mechanism involved in the genesis NOMI which refers to acute mesenteric ischemia without occlusion of the mesenteric arteries (**Figure 2**). (17, 18) Tissue damage from NOMI usually begins with mesenteric vasospasm, however, blood flow restoration (such as during DKA fluid treatment) exacerbates injury due to ischemia/reperfusion. (19) After the onset of ischemia, three different processes may be distinguished: the ischemic phase, reperfusion and the injury phase. In acute arterial occlusion these processes occur sequentially; in non-occlusive ischemia different stages can occur simultaneously, intermittently, or even repeatedly. The re-establishment of oxygenated blood flow produces oxygen-free radical metabolites with local inflammation in previously hyperperfused regions of the intestine. Cell damage induced by prolonged ischemia-reperfusion injury may lead to apoptosis, autophagy, necrosis, and necroptosis. (20) The integrity of the membrane is lost, with further cell necrosis. Not only is the damaging effect of Reactive Oxygen Species (ROS) greater than that of pure ischemia, but the damage is also no longer limited to the ischemic area. (21) NOMI accounts for about 20-30% of all cases of mesenteric ischemia and represents a potentially lethal condition with mortality rates up to 50%. (17, 22–25)

### NOMI as Complication of DKA

It tends to occur mostly in elderly patients who have low-cardiac output and other risk factors, but several pediatric cases have been previously described. These children usually present with underlying diseases such as familial dysautonomia, Addison’s disease, situs inversus, burns, chemotherapy for hematological malignancies, encephalitis, and septic shock. (26–34) Although rare, NOMI is a complication of DKA observed in adult diabetic patients. (18) Most described cases are patients older than 50 years of age and their rapid worsening condition required surgery to remove necrotizing intestine. (35–39) Two pediatric cases of NOMI in the context of DKA have been documented (**Table 2**). (40, 41) The general clinical conditions and abdominal symptoms of these patients, despite adequate fluid resuscitation and insulin administration, suggest a concurrent complicating event. When distinctive signs of ischemia were revealed during imaging assessment, both children underwent laparotomy and subsequent intestinal resection. The clinical evolution of the adolescent male described by Chan Chua et al. was probably driven by underlying neurological comorbidities (autonomic neuropathy). (41) Presently, the 3-year-old girl described by Di Meglio et al. is the only reported case of NOMI in a previously healthy child at T1D onset. (40) The pathogenesis of NOMI in DKA remains elusive although the two conditions most probably involve at least partially shared underlying mechanisms. Several previous studies have suggested that mesenteric artery spasm may be promoted by catecholamine, renin-angiotensin system and vasopressin activation during DKA. (42) Nieuwdorp M et al. have also suggested that endothelial glycocalyx (EG) dysfunction may be one of the possible mechanisms of NOMI onset in DKA. (43) EG is a layer of proteoglycans that covers the vascular endothelium, consisting of a core protein that carries one or more covalently attached glycosaminoglycan chains; it regulates vascular tone and permeability, inflammation and coagulation. (18, 44) EG damage during hyperglycemic conditions may cause vasospasm, hypoperfusion, as well as promoting inflammation and microthrombi formation due to hypercoagulability, ultimately resulting in NOMI. (18)

**Table 2:** Summary of pediatric DKA-related NOMI in literature.

	*Sex*	*Age(years)*	*Underlying diseases*	*Degree of DKA (ph)*	*Clinical presentation*	*Treatment of DKA*	*Treatment of NOMI*	*Outcome*
Di meglio et al.(40)	Female	3	None	6.99	Classical signs of DKA* Diffuse abdominal pain, single episode of vomiting diminished bowel sounds	intravenous fluids (20 mL/kg of 0.9% NaCl) insulin infusion (0.05 U/kg/h)	Laparotomic intestinal resection (from the distal jejunum to the right transverse colon) Antibiotics total parenteral nutrition	No post-surgery complication Ileostomy (4 months later) resumption of complete enteral feeding at 3 months
Chan-Chua et al(41)	Male	17	Poorly controlled MDI-treated T1DM (autonomic neuropathy vascular damage) short stature, hepatomegaly delayed puberty	6.92	Dehydration, Kussmaul breath leg cramps diffuse abdominal pain (60 hour later) fever	intravenous fluids (1500 mL of lactated Ringer and 0.45% NaCl) regular insulin sc (25 U) sodium bicarbonate ev (100 mEq)	Laparotomic intestinal resection (terminal ileum and single abscess formation)	No complication
Frontino et al.	Female	13	T1DM treated with SAP	6.91	Dehydration, Kussmaul breath GCS 12/15 Incoerciblevomiting oligo-anuria (AKI) Modest abdominal pain (48 hours later) loose stools fever Clostridium difficile infection	Intravenous fluids (20 mL/kg of 0.9% NaCl) Insulin infusion (0.1 U/kg/h)	Laparotomic intestinal resection (44 cm of ileum) Antibiotics total parenteral nutrition	No post-surgery complication Resumption of complete enteral feeding at 3 weeks

*(lethargy, polyuria, polydipsia, anorexia, dehydration).

## Conclusion

Despite the use of technology, recurrent DKA prevention remains an unmet need in children and adolescents with T1D and may be accompanied by life-threatening acute complications. The diagnosis of DKA-associated NOMI must be suspected in pediatric patients with DKA, persistent abdominal pain, and severe dehydration. Importantly, our case underlines how NOMI may exhibit a very subtle onset with mildly progressive abdominal pain, and unremarkable ultrasound imaging over days until overt symptoms and signs of complications (ie. Intestinal perforation) manifest.

## Data Availability Statement

The original contributions presented in the study are included in the article/supplementary material. Further inquiries can be directed to the corresponding author.

## Ethics Statement

Ethical review and approval was not required for the study on human participants in accordance with the local legislation and institutional requirements. Written informed consent to participate in this study was provided by the participants’ legal guardian/next of kin.

## Author Contributions

GF and RT share first authorship due to equal contribution. GF, RT and VC reviewed the case data and literature and contributed to manuscript drafting. GF and RT are responsible for final revision of the manuscript and significant intellectual content. All authors issued final approval for the version to be submitted. All authors contributed to the article and approved the submitted version.

## Conflict of Interest

The authors declare that the research was conducted in the absence of any commercial or financial relationships that could be construed as a potential conflict of interest.

## Publisher’s Note

All claims expressed in this article are solely those of the authors and do not necessarily represent those of their affiliated organizations, or those of the publisher, the editors and the reviewers. Any product that may be evaluated in this article, or claim that may be made by its manufacturer, is not guaranteed or endorsed by the publisher.
